# The impacts of economy policy uncertainty on peer effects of firms R&D investment: Based on LDA machine learning and regression statistical modeling approach

**DOI:** 10.1371/journal.pone.0305715

**Published:** 2024-06-24

**Authors:** Shanshan Wang, Xingxing Yang, Xia Zhu, Xiangyu Ge

**Affiliations:** 1 School of Statistics and Big Data, Henan University of Economics and Law, Zhengzhou, China; 2 School of Finance and Economic, Wuhan College, Wuhan, China; 3 School of Statistics and Mathematics, Zhongnan University of Economics and Law, Wuhan, China; 4 Department of Finance, Wuhan Technology and Business University, Wuhan, China; Universidad de Salamanca, SPAIN

## Abstract

The Latent Dirichlet Allocation (LDA) model is used to extract the text themes of newspaper news and construct the Chinese Economic Policy Uncertainty (EPU) Index. On this basis, based on the relevant data of Chinese A-share listed companies from 2008 to 2020, this paper empirically analyzes the impact of EPU on peer effects of firms R&D investment, and finds that EPU will aggravate the peer effects of firms R&D investment. Furthermore, the moderating effect of manager’s motivation to maintain reputation on the process of EPU influencing the peer effects of firms R&D investment was tested, and the mechanism of EPU influencing the peer effects of firms R&D investment through financial frictions was verified.

## 1. Introduction

The government routinely intervenes in the economy by formulating and adjusting economic policies. Since the financial crisis, China has successively introduced a series of macroeconomic policies, such as "CNY 4 Trillion Stimulus Plan", "Strategic Emerging Industry Revitalization Plan", "Belt and Road Initiative", "Internet +", "Lowering Reserve Requirements and Interest Rates", "Mass Entrepreneurship, Mass Innovation", "Made in China 2025" to deal with the economic recession and market contraction. In order to speed up the economic recovery after the pandemic, the Chinese government has successively issued a series of counter-cyclical economic regulation policies, and continuously adjusted the policies with the changes of the pandemic at home and abroad. The introduction and implementation of these policies have reduced the negative impact of the global economic downturn and pandemic outbreak on China’s economy to a certain extent. At the same time, however, the government’s active exploration of economic intervention has also brought a high degree of economic policy uncertainty.

Compared with economic policy itself, the impacts of EPU is more hidden but not to be underestimated. Therefore, the research on the consequences of EPU has been widely concerned by scholars. The R&D investment behavior of companies is a strategic investment decision-making behavior that directly affects and plays a decisive role in the improvement of their independent innovation ability. Accordingly, under the policy background of mass innovation and mass entrepreneurship, the research on R&D decision-making behavior of companies as the main body of market activities is still of great value. The existing literature has generally carried out the research on the impacts of EPU on company R&D innovation from two perspectives. First, based on the growth option theory or the research from the perspective of R&D investment income (see Meng & Shi [1]), it is considered that the rise of the level of EPU will improve the expected income of technological innovation, which will promote companies to increase R&D investment. Second, the research based on the real option theory or the perspective of R&D investment cost provides the opposite conclusion (see Hao et al. [2], Xu [3]). Thus, there is no consistent conclusion about the impacts of EPU on company technological innovation, and most studies are carried out from the direct impact of economic policy uncertainty on individual R&D investment, and seldom consider the learning and imitation between companies in the decision-making process of R&D investment, and then there are few studies on the indirect impacts of EPU on company R&D investment from the perspective of peer effects. According to social interaction theory was studied by Rabin [4] and social learning theory provided by Zeckhauser et al. [5], companies are not completely independent in the decision-making process, and will be affected by corporate decisions related to the same industry, region or other aspects. Previous studies have shown that companies have peer effect in multiple aspects, such as investment decision by Bustamante & Frésard [6], capital structure by Lian et al. [7], dividend policy by Grennan [8] and Wang et al. [9], corporate social responsibility by Li & Wang [10], cash holding by Machokoto et al. [11] and Zhuang et al. [12], management forecast by Seo [13] and Machokoto et al. [14], tax evasion by Gao et al. [15], innovation investment by Xiao et al. [16]. However, there is almost no research on the peer effects of R&D investment in the external environment of EPU from the perspective of behavioral economics.

In addition, in the selection of economic policy uncertainty index, most of the literature directly use the Chinese EPU index constructed by Baker et al. [17], Huang & Luk [18]. Baker et al. [17] used the *South China Morning Post* as the text source to construct the index through keyword screening, statistics, index standardization and normalization processing. However, there is a big difference in expression between English and Chinese, and there may be a certain degree of one-sidedness in using only one Hong Kong newspaper to reflect the situation in the mainland. Huang & Luk [18] adopted a similar index construction method, except that the news texts used came from 10 mainland newspapers and periodicals, such as *Beijing Youth Daily*, and compiled the Chinese keywords of the corresponding Chinese EPU index. Even so, keyword search method is employed to screen news texts in the process of index measurement, while the comprehensiveness of policy keyword coverage and the accuracy of topic generalization are poor, and the construction of keyword sets depends on manual tagging. With the continuous change of the economic situation, it is necessary to spend high labor costs to update the keyword set. This work draws lessons from Azqueta-Gavaldon [19] to reconstruct the EPU index suitable for China. On the one hand, the text messages of the three official authoritative newspapers from the mainland, *Economic Daily*, *Nanfang Daily* and *Guangming Daily*, have been included, and their authority and comprehensiveness make them an important source of information for participants in China’s economic market. On the other hand, LDA model by Blei et al. [20] is used to analyze the topic of news, which to some extent makes up for the shortcomings of incomplete keyword search and inaccurate topic determination, and alleviates the problem of high cost and inefficiency of manually updating keyword sets.

Accordingly, taking Chinese A-share listed companies in Shanghai and Shenzhen stock markets from 2009 to 2020 as samples, using the constructed Chinese EPU index, this paper empirically analyzes the impact of EPU on corporate R&D investment peer effect. Furthermore, it examines the regulatory role of managers’ reputation motivation in the process of EPU affecting R&D investment peer effects, and verifies the mechanism of the impacts of EPU on R&D investment peer effects through financial friction.

The contributions of this work include:

First, the existing literature on the influencing factors of firm R&D investment is mostly from the perspective of individual companies, resulting in that the interaction between companies is not taken into account, which enriches the analysis of the influencing factors of firm R&D investment;

Second, the classical measurement method of index is improved to re-measure Chinese EPU index. Moreover, the applications of text analysis and machine learning algorithms in economic problems have been enriched;

Third, the existing literature mainly analyzes the direct impacts of EPU on company R&D investment, and this work attempts to explore the "black box" problem of EPU affecting R&D investment behavior with the help of peer effect as an indirect channel. Simultaneously, this work creatively explores the possible mechanism of the impacts of EPU on R&D investment peer effects from the perspectives of managers’ reputation motivation and financial friction. Therefore, it not only enriches the research of EPU, but also further expands the related research of firm R&D investment in peer effects.

This paper is arranged as follows. The EPU index measurement based on LDA machine learning method are presented in Section 2. The theoretical analysis and research hypothesis are presented in Section 3. The modeling design is listed in Section 4. The empirical research in terms of empirical results analysis, endogeneity test and robustness test are correlated, and the impacts of EPU on peer effects of firm R&D investment is given in Section 5. The regression statistical models are used to further examination the influence mechanism of EPUs on peer effects of firm R&D investment in Section 6, and conclusions are offered in Section 7.

## 2. EPU index measurement based on LDA machine learning method

**Fig 1:**
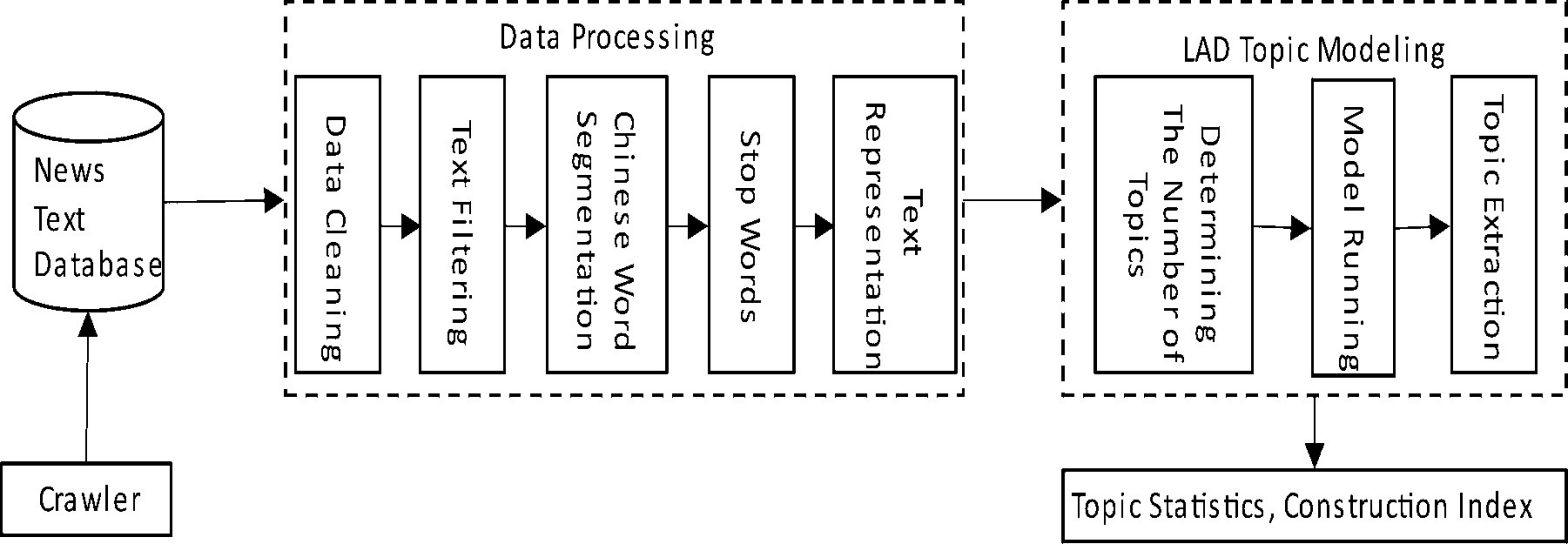
Schematic diagram of EPU index measurement framework.

The construction of EPU index mainly includes news text data collection, data processing, LDA topic modeling, related topic statistics and index construction (see Fig 1). The details are as follows:

### 2.1. Data acquisition

A Python crawler program is compiled, and a large number of news articles are collected to form a text database with newspapers as crawling objects. In fact, most investors look at the market and the economy through news and analytical reports. Based on the investigation of the authority and text availability of domestic newspapers and periodicals, the text data are mainly from the official websites of the three official authoritative newspapers of Chinese mainland, namely, *Economic Daily*, *Nanfang Daily* and *Guangming Daily*. When obtaining the text data of newspaper news, the Python crawler framework is established by using Scrapy crawler technology to grab the text information of all news from the website. As the earlier historical data could not be obtained, the news texts from January 1, 2008 to December 30, 2020 were crawled, and the missing data of individual dates were obtained manually through the China knowledge Network newspaper database. The crawled newspaper data is stored in a file in CSV format, which contains eight field names, namely, newspaper name, date, layout number, layout name, news serial number, news title, news link and text content of the news.

### 2.2. Data processing

The obtained text data is processed, including data cleaning, text filtering, Chinese word segmentation, removal of stop words and text representation, and the dictionary and corpus that can be used for subsequent LDA model analysis are obtained. Among them, data cleaning refers to the deletion of duplicate data, noise data and too little text data. After collation, the newspaper database contains 275705 items of news from *Economic Daily*, 642386 pieces of news from *Nanfang Daily* and 247350 items from *Guangming Daily*, totaling 1165441 items.

Text screening refers to the screening of texts containing economic keywords and uncertain keywords. Referring to the research of Huang & Luk [18], the keywords of economic category and uncertain category are determined, as shown in Table 1. First of all, the data of three kinds of newspapers after data cleaning are merged. Then, based on the merged newspaper text data, all the news containing economic keywords are screened according to the keywords in Table 1. Finally, on the basis of the news containing economic keywords, we further screen all the news containing uncertain keywords. From January 2008 to December 2020, the total number of news items containing economic terms was 440938, while the total number of news items containing both economic and uncertain terms was 39778.

**Table 1:** Keywords for China’s economic category and uncertainty category.

Category	
Economics	"Economy", "Finance"
Uncertainty	"uncertain", "unsure", "fluctuating", "vague", "doubtful", "concussive", "turbulent", "unstable", "mysterious", "hesitant", "foreign", "unknown", "indefinite", "unidentified", "uncertain", "unclear", "undecided", "unpredictable", "volatile" "changeable", "random"

Subsequently, the jieba Chinese word segmentation package is used to segment the text database of the above newspaper news under the framework of Keras, and the commonly used "Harbin University of Technology stop word list", "Baidu stop word list" and "Sichuan University Machine Intelligence Laboratory stop word list" are integrated to get the basic stop word list and remove the stop words in the text data. With the help of the relevant commands in python language, we can get the dictionary that can be used for subsequent LDA model analysis, and the word bag model that can be recognized by computer, namely corpus.

### 2.3. LDA topic modeling

This part uses the genism toolkit based on python language to train the LDA model. The development environment is Jupyter Notebook, which mainly includes the following two steps:

#### Step 1: Determine the optimal number of topics and run the model

**Fig 2:**
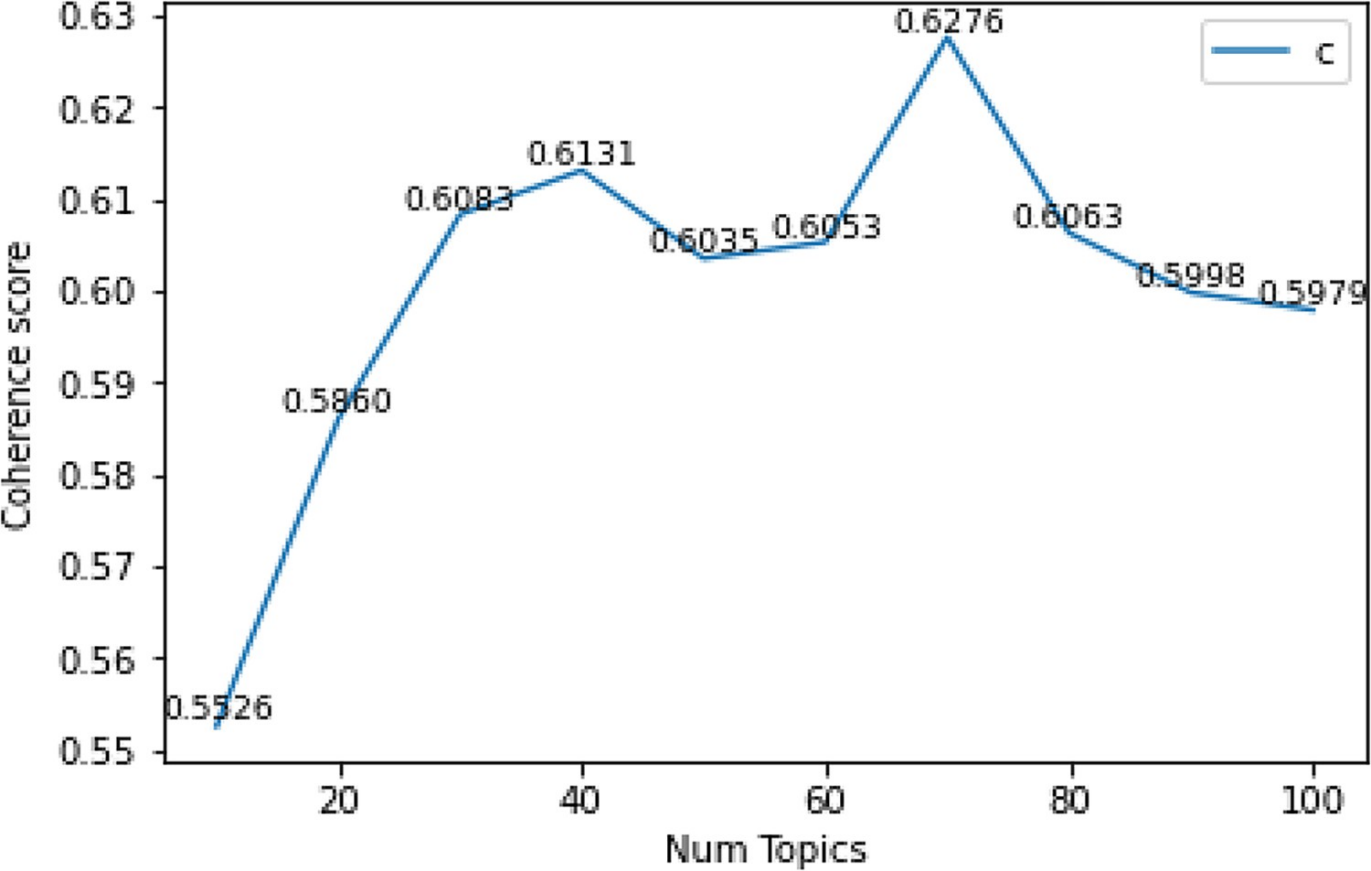
Degree of consistency under different topics.

In the process of building the LDA model, the number of implied topics needs to be determined in advance, and whether the number of topics is reasonable will directly have an important impact on the quality of the model (see Gan & Qi [21]). Topic consistency is the most effective method to measure the quality of topics, and it is also one of the important techniques to estimate the number of topics (see Stevens et al. [22]). The method to find the optimal number of topics by using topic consistency is to build multiple LDA models with different number of topics and select the number of topics corresponding to a LDA model that provides the highest consistency value. In this work, taking the topic consistency index as the criterion, the appropriate number of topics is selected by experimental method, and the experimental results are shown in Fig 2. Obviously, when the number of topics is 70, the semantic similarity of words in the topic is the highest, and the highest score of topic consistency is 0.6276, so the optimal number of topics is 70.

#### Step 2: LDA model to run and extract news document topics

In the running result of the LDA model, each document has a corresponding document-topic distribution, that is, each document is assigned to multiple related topics, and each topic corresponds to a certain probability. The topic with the highest probability is selected as the most representative topic of the document, and the document is tagged, that is, only one topic is given to each document. Furthermore, combined with the topic keywords and the corresponding news document content, the generated 70 topics are summarized. For example, when the subject words correspond to finance, expenditure, local, capital, government, budget, etc., and vocabulary, the theme of the document can be defined as government expenditure within the scope of fiscal policy. A total of 26 policy-related themes are selected, such as fiscal policy, monetary policy, trade policy, exchange rate policy, industrial policy and policy regulation. As shown in Table 2, the list on the left shows the subdivided topics summarized, and the list on the right shows the keywords corresponding to each topic trained by the model, and only the first 10 are shown here.

**Table 2:** Display of news topic results.

Theme	Extracted subject words (top 10)
Government expenditure	Finance, expenditure, local, funds, government, budget, central, local government, arrangements, revenue
Tax policy	Policy, introduction, government, subsidy, tax, collection, cost, support, reduction, implementation
macro-control	Investment, growth, macro-control, policy, employment, structure, stability, stability, increase, strength
Public project engineering	Project, construction, planning, center, transformation, engineering, development, city-wide, supporting, transportation
Consumption and investment	Consumption, growth, income, GDP, investment, improvement, economic growth, per capita, residents, demand
Pro-agricultural policy	Villagers, poverty alleviation, poverty alleviation, masses, work, assistance, disaster areas, villages, villages, rural areas
National defense and military	Military, army, rescue, strategy, country, war, army, national defense, combat, strength
Currency loan policy	Central bank, interest rate, loan, monetary policy, bank, liquidity, credit, deposit, interest rate cut, deposit reserve ratio
Price regulation and control	Prices, increases, CPI, inflation, prices, increases, price increases, food, impacts, factors
Trade export Open to the outside world	President, Xi Jinping, Cooperation, Belt and Road Initiative, leaders, country, Prime Minister, Relations, President, present
	Cooperation, countries, promotion, areas, trade, relations, leaders, summits, international, BRICS
	Exports, trade, foreign trade, growth, import and export, international, decline, orders, processing trade
	World, international, peace, mankind, global, country, people, cooperation, promotion, opening up
	International, global, investment, opening up, overseas, forum, mainland, foreign investment, domestic, national
Exchange rate policy	Dollar, Gold, Gold Price, Federal Reserve, interest rate hike, Investor, Euro, International, Global, Investment
	CNY, exchange rate, USD, CNY exchange rate, appreciation, currency, depreciation, foreign exchange, hot money, foreign exchange reserves
Environmental regulation	Ecology, environment, pollution, discharge, protection, control, disasters, regions, climate change, engineering
Industrial transformation	Industry, transformation, upgrading, development, industry, Pearl River Delta, development, manufacturing, resources, acceleration
Innovation and entrepreneurship	Innovation, science and technology, research and development, country, research, talent, field, investment, independent innovation
	Services, platforms, innovation, models, data, provision, Internet, promotion, entrepreneurship, areas
structural reform	Reform, innovation, promotion, development, promotion, institutional, economic development, acceleration, strategy, supply
Emergency prevention	Pandemic, Prevention and Control, Influenza, Emergency, pneumonia, recovery, COVID-19, return to work, supplies, Global
	Risk, ability, response, stability, change, improvement, challenge, international, prevention, situation
Legal supervision	Laws, protection, legislation, systems, regulations, perfection, rule of law, intellectual property rights, protection, regulations
Government supervision	People, history, nation, general secretary, Chinese nation, reform and opening up, spirit, times, Xi Jinping, rejuvenation
	State, politics, nationality, president, election, West, people, ethnic minorities, government, support

### 2.4. Construction of economic policy uncertainty index

Targeted statistics of the distribution of related topics and the construction of EPU index (see Table 2).

The first step is to count the number of articles on each topic each month for the 26 topics related to EPU, and then get 26 original time series (The Chinese EPU index measured by Baker et al. [17] and others is obtained by dividing the number of target news by the number of all news, while the index constructed by Huang & Luk [18] is obtained by dividing the number of target news by the number of news only related to the economy. The latter is better than the former. Many news articles have nothing to do with the economy, and the number and types of pages that are not related to economic policy will change over time, except for the main layout categories.

Therefore, including it in the denominator of the target news frequency calculation formula will enable the result of the measure unreasonable to a certain extent). Since the total number of articles varies with time, divide each original time series by the total number of articles containing "economic" keywords each month to get a time series consisting of the monthly frequency of 26 topics, recorded as *X*_*it*_, *i* = 1,2,⋯,26, *t* represents the month.

In the second step, the standardized frequency series corresponding to 26 topics is summed up by month to get the monthly time series *Y*_*t*_, *t* represents the month. The monthly time series is standardized according to the standard deviation from 2008 to 2020. Specifically, the variance $\sigma_{Y}^{2}$ of *Y*_*t*_ in the period from January 2008 to December 2020 is calculated, and then the *Y*_t_ is standardized to get *Z*_*t*_ = *Y*_*t*_/*σ*_*Y*_, *t* for all months.

The third step is to standardize the sequence *Y*_*t*_ to an average of 100 according to the following Eqs (1) and (2).

$$M=\frac{\sum_{t=1}^{N}Z_{t}}{N}$$ (1)

$$\mathrm{EPU}=Z_{t}•\frac{100}{M}$$ (2)

Wherein, *N* represents the number of months in the sample time interval, *M* represents the monthly average of *Z*_*t*_ and the final time series is the EPU index.

### 2.5. Analysis of the characteristics of exponential fluctuation

**Fig 3:**
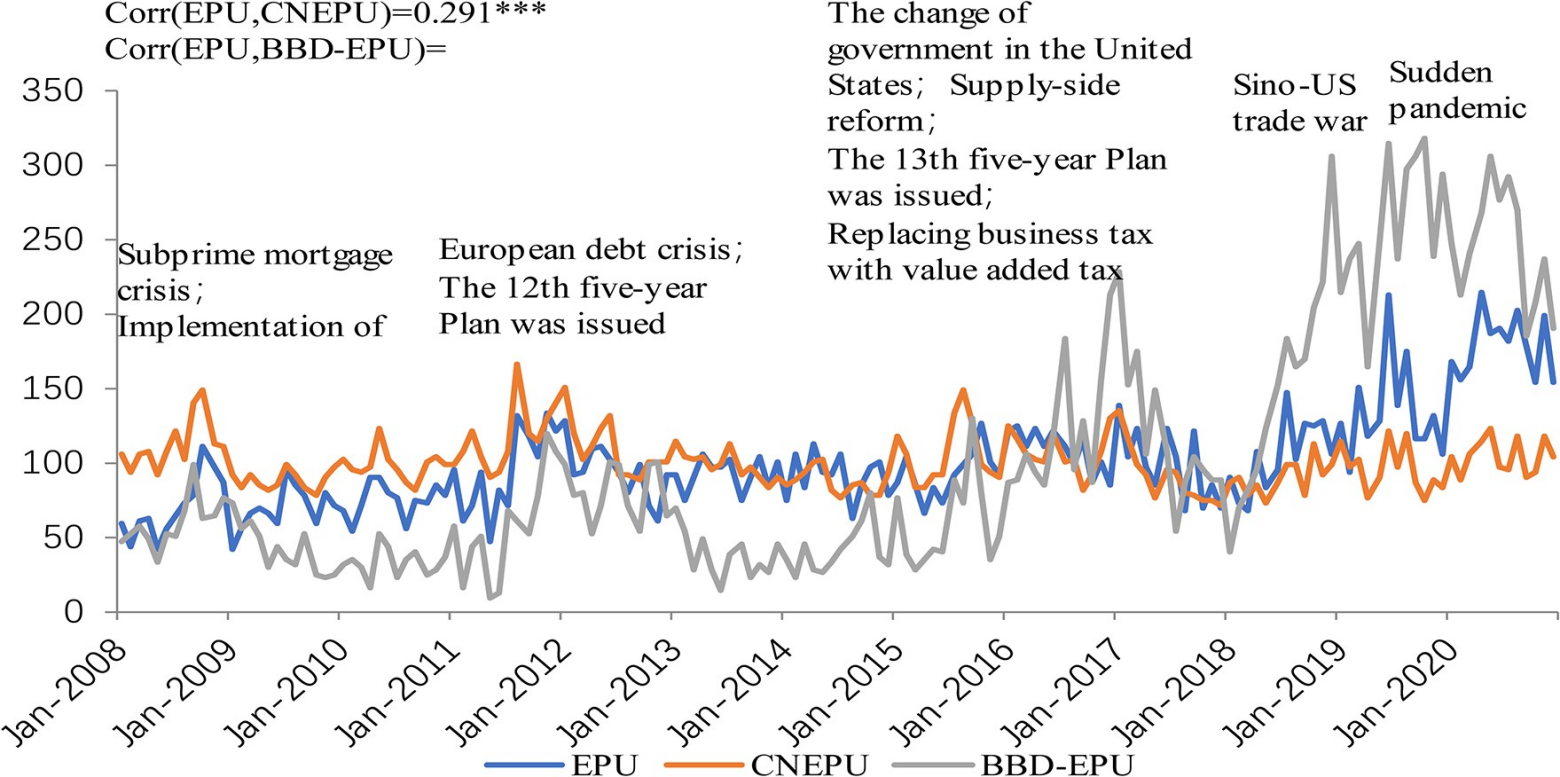
EPU index and other uncertainty index measurement. Notes: In order to facilitate the comparison, the time series corresponding to the two indices are also standardized to a standard deviation and an average of 100 during the sample period.

The volatility of the measured EPU index and the existing EPU index is provided by Fig 3.

The overall change trend and fluctuation peak and trough of Chinese EPU (CNEPU) index are consistent with the actual economic development and economic events, and the joint action of domestic factors and international environment leads to the fluctuation of CNEPU. On the other hand, the correlation between the measured EPU index and the CNEPU index constructed by Huang & Luk [18] is 0.291, which is significant at 1% level, and the correlation with the BBD-EPU index constructed by Baker et al. [17] is 0.742, indicating that the fluctuation characteristics of the measured EPU index are similar to those of the existing index as a whole. These two aspects basically show that the measured results of EPU index are reasonable.

In addition, there are differences among the three indices. On the whole, the volatility of the BBD-EPU index is more extreme, while the CNEPU index is more stable. In particular, near the end of the sample period, due to the impact of the COVID-19 pandemic on all aspects of China’s economy, the uncertainty index will increase. During this period, the measured EPU index fluctuated between the two existing indices, which relatively reasonably reflected the fact that the pandemic had an impact on China’s economy.

## 3. Research hypothesis

Peer effects means that an individual will be influenced by a group decision with similar characteristics when making a decision. Lieberman & Asaba [23] argued that in order to obtain useful decision-making information and maintain its advantage in market competition, enterprises will strengthen their learning and imitation with other similar enterprises, thus forming the source of peer effects. For the R&D investment activities of enterprises, R&D activities are a kind of exploratory behavior of innovation in unknown areas, which has the characteristics of long cycle, high cost and high technological uncertainty. Once the innovation is not successful, it will cause great irreversible losses to the enterprise. Furthermore, the ultimate goal of R&D investment activities of listed companies is to make economic profits in the market. Even if the technical level is successful, there are still great risks in the process of transforming technology into economic benefits. In order to make the technological R&D achievements into economic profits smoothly, listed companies need to accurately predict the future market space and their position in the future market. However, the speed of product upgrading is fast, there are higher requirements for the innovation of enterprise R&D technology, the internal market environment and external policy environment faced by enterprises have a large degree of uncertainty, and the national legislation on enterprise innovation patent and intellectual property protection is not perfect. Therefore, the R&D investment of enterprises is a complex and high-tech financial decision, which is highly dependent on information, and it will not be comprehensive enough to use only the information they have. Enterprises in the same industry and focus enterprises face the same external market and policy environment, and their R&D investment decision can convey information about the development opportunities and market predictions of the industry, which is of great reference value for focus enterprises. As a result, enterprises have a strong willingness to refer to and imitate the R&D behavior of enterprises in the same industry in order to shorten the R&D trial and error process as much as possible, reduce the risk of R&D investment, and improve the conversion rate of economic benefits.

On the other hand, R&D activities are an important means for enterprises to participate in market competition and a booster for enterprises to enhance their economic value in the future. Compared with the traditional investment activities, the results of R&D activities have a greater impact on the future competitive advantage and market position of enterprises. For the focus enterprises, the R&D investment intensity of the same group enterprises can convey the overall innovation willingness intensity of the same industry enterprises, and the main R&D investment areas chosen by the same group enterprises is a kind of enlightenment about the future new market development direction. There is technological competition among enterprises in the same industry. Therefore, enterprises will learn and learn from the intensity and trend of R&D investment of peer enterprises in order to maintain or improve their market position. When the enterprises in the same group increase their R&D investment, the focus enterprises tend to take measures to improve the level of R&D investment, so as not to lag behind, that is, enterprises will adjust their decisions according to the investment decision of competitors (see Chen et al. [24])^.^ Based on the analysis, hypothesis 1 is put forward.

**Hypothesis 1**: there is an industry-based peer effects in R&D investment of Chinese listed enterprises.

The EPU will change the business environment of enterprises, and the overall risks related to policies will inevitably directly or indirectly affect the business activities of enterprises. With the enhancement of EPU, the degree of incompleteness and asymmetry of information obtained by enterprises increases. The investment cycle of R&D activities is long and the risk is high. Whether the results can be transformed into enterprise profits depends on the future market demand. In the case of frequent economic policy adjustments, it is more difficult for enterprises to accurately judge the demand of new markets. At this time, managers will adopt risk-averse strategy and are more willing to learn and learn from the information implied in R&D investment decision of other enterprises. Homogenization strategy is adopted to match the behavior of competitors, reduce the intensity of competition or reduce risk, and finally show a greater degree of imitative investment. On the other hand, the enhancement of EPU leads to the conflict between the large amount of information needed by managers to make reasonable judgments and the high cost of information acquisition. Therefore, in order to deal with the supervision of stakeholders, managers will be more inclined to rely on public information and try to keep up with the average standards of most similar enterprises in the same industry, which further deepens the peer effects of R&D investment. Based on the above analysis, hypothesis 2 is put forward.

**Hypothesis 2:** EPU will further promote the peer effects of R&D investment.

## 4. Modeling design

### 4.1. Data source

The Ministry of Finance of the People’s Republic of China issued the *Accounting Standards for Companies—Intangible Assets* in 2006, which requires companies to disclose research and development expenditures, and this regulation has been implemented on January 1, 2007. In view of the implementation of the new accounting standards, the availability and credibility of the data, Chinese A-share listed companies in Shanghai and Shenzhen stock markets from 2009 to 2020 are selected as the research object. Some variables need to be dealt with one period lag, so the initial sample interval is from 2008 to 2020. After obtaining the sample data, the following screening processing is carried out:

- the samples of the financial industry and utilities are removed;
- the samples of abnormal financial system during the sample period (ST, * ST, PT) are removed;
- the samples with incomplete financial data are removed;
- the samples with less than 5 enterprises in the same industry are removed; and
- in order to eliminate the influence of extreme values, data other than 1% and 99% quantiles of continuous variables are tailed with Winsor.

The variable index data at the enterprise level come from Guotai’an Research Service Center (CSMAR series database) and China Research Data Service platform (CNRDS platform). The data of related variables of enterprises in the same group are calculated. According to the CSRC "guidelines on Industry Classification of listed companies" (2012 edition) as the industry classification standard, the classification of the industry determines the division of enterprises in the same group. In order to avoid the lack of samples or too many enterprises in the same industry, the manufacturing industry is classified according to the second-level code and other industries are classified according to the first-level code.

### 4.2. Index selection

Selection 1: R&D investment (*RD*_*t*_). Due to the large differences in the asset scale of enterprises at different stages of development, in order to eliminate the impact of this difference, the R&D investment intensity is used to measure the R&D investment status of enterprises. According to the two methods commonly used in existing research, the intensity of R&D investment is calculated: one is the proportion of R&D investment to operating income (*RD*1_*t*_), and the other is the proportion of R&D investment to total assets (*RD*2_*t*_).

Selection 2: R&D investment by peer enterprises (*PeerRD*_*t*−1_). Drawing lessons from Leary & Roberts [25] and Peng et al. [26], it is measured by the average R&D investment intensity of other enterprises in the same industry excluding this enterprise, that is, the calculation formula of *PeerRDin*_−*ijt*−1_ is:

$$PeerRD_{-ijt-1}=\frac{\sum_{m=1(m\neq i)}^{N}RD_{mji-1}}{N-1}$$ (3)

Among them, *RD*_*mji*−1_ indicates the R&D investment intensity of company *m* belonging to industry *j* in *t*−1 year, and *m*≠*i* indicates that when calculating the R&D investment level of peer companies of Focus Company *j*, the R&D investment data of Focus Company *i* itself is removed. *N* represents the number of companies in the industry where Focus Company *i* is located.

Selection 3: EPU Index (*EPU*_*t*−1_): An economic policy uncertainty index measured using the previous LDA machine learning methodology. Given that the index is monthly, in actual use, the index of the monthly frequency is converted into an annual index by arithmetic average, and the annual index is divided by 100 to adjust by orders of magnitude (see Meng & Shi [1]).

Selection 4: Control variables. Referring to the existing research results, the cash-to-asset ratio (*Cash*), asset-liability ratio (*Lev*), profitability (*Roa*), company value (*TobinQ*), tangible asset ratio (*Tangible*), company age (*Age*) and size (*Size*) were selected as the control variables. At the same time, drawing on the relevant research of company peer effect, the industry average of the above variables is also controlled (consistent with the calculation method of (*PeerRD*).

Selection 5: Macroeconomic variables (*M*_*t*_). As the adjustment of monetary policy will affect the financial situation and financing capacity of companiew, and entrepreneurs’ feelin gs and confidence in the macroeconomic environment will affect the R&D investment behavior of companiew. Therefore, drawing lessons from Wang et al. [27], two macroeconomic indicators, money supply (ln*M*2_*t*_) and entrepreneur confidence index (*eei*_*t*_), are selected to control the characteristics of the period.

The variables involved in the model and their definitions are shown in Table 3.

**Table 3:** Definition of variables and specific calculation methods.

Variables	Variable symbol	Variable definition
Explained variables		
Research and development investment	*RD*1_*t*_	Total R&D investment / operating income
	*RD*2_*t*_	Total R&D investment / total assets
**Explanatory variable (lag one period)**		
R&D investment of peer companiew	*P*eer*RD*1_*t*−1_	Average of other companiew in the same industry *RD*1_*t*−1_
	*P*eer*RD*2_*t*−1_	Average of other companiew in the same industry *RD*2_*t*−1_
Economic policy uncertainty index	*EPU*_*t*−1_	See the previous section for details
**Control variable (lag one period)**		
Cash asset ratio	*Cash*_*t*−1_	Cash and cash equivalents balance / total assets
Asset-liability ratio	*Lev*_*t*−1_	Total liabilities / total assets
Company profitability	*Roa*_*t*−1_	Total net profit / assets
Company value	*TobinQ*_*t*−1_	Market capitalization / total assets
Tangible assets ratio	*Tangible*_*t*−1_	Total tangible assets / total assets
Age	*Age*_*t*−1_	Ln (year of study-year of listing + 1)
Company scale	*Size*_*t*−1_	Natural logarithm of total assets
Cash asset ratio of peer companiew	*PeerCash*_*t*−1_	Average cash asset ratio of other companiew in the same industry
Asset-liability ratio of peer companiew	*PeerLev*_*t*−1_	Average asset-liability ratio of other companiew in the same industry
Profitability of peer companiew	*PeerRoa*_*t*−1_	Average profitability of other companiew in the same industry
Peer company value	*PeerTobinQ*_*t*−1_	Average value of other companiew in the same industry
Ratio of tangible assets of peer companiew	*PeerTangible*_*t*−1_	Average tangible assets ratio of other companiew in the same industry
Age of peer company	*PeerAge*_*t*−1_	Average age of other companiew in the same industry
Scale of peer companiew	*PeerSize*_*t*−1_	Average company size of other companiew in the same industry
**Macroeconomic variables**		
Money supply	ln*M*2_*t*_	The logarithm of annual broad money supply M2
Entrepreneur confidence index	*eei*_*t*_	Data from the Company Prosperity Survey conducted by the National Bureau of Statistics

### 4.3. Model building

#### 4.3.1. Peer effects of R&D investment in listed companies

With reference to the research ideas of Leary & Roberts [25], the following models are constructed to test the existence of R&D investment peer effect (see Hypothesis 1).


$$RD_{ijt}=\alpha +\beta PeerRD_{-ijt-1}+\gamma_{1}Controls_{ijt-1}^{Firm}+\gamma_{2}Controls_{-ijt-1}^{Peer}+\mu_{i}+\nu_{t}+\varepsilon_{it}$$ (4)

In model (4), *i*, *j*, and *t* represent the individual, industry, and year, respectively. *RD*_*ijt*_ represents the intensity of R&D investment in the t year of the company *i* in industry *j*; *PeerRD D*_−*ijt*−1_ represents the average R&D investment intensity of the same group of companies in the *t*−1 year of the focus company *i*; *-i* means that when calculating the average R&D investment of the same group of companies of the focus company *i*, the R&D investment data of the company *i* itself is removed; $Controls_{ijt-1}^{Firm}$ represents the control variable at the company level; $Controls_{-ijt-1}^{Peer}$ indicates the corresponding control variables at the level of cohort firms, that is, the R&D investment of companies may also be affected by the financial characteristics of other companies in the same industry; *μ*_*i*_ is used to control the timeless influence of individual companies, which can reduce the impact of some unobservable individual heterogeneity to a certain extent; *v*_*t*_ indicates that the year is fixed to control the influence of time trends, and finally a two-way fixed-effect model; *ε*_*it*_ indicates the random distractor. The positive and negative coefficients of *β* and the significance are used to determine whether there is a peer effect in the R&D investment of Chinese listed companiew.

#### 4.3.2. Peer effects between EPU and company R&D investment


$$\begin{array}{c} RD_{ijt}=\alpha +\beta_{1}PeerRD_{-ijt-1}+\beta_{2}PeerRD_{-ijt-1}\times EPU_{t-1}+\beta_{3}EPU_{t-1}\\ +\gamma_{1}Controls_{ijt-1}^{Firm}+\gamma_{2}Controls_{-ijt-1}^{Peer}+\mu_{i}+\theta M_{t}+\varepsilon_{it} \end{array}$$ (5)

In model (5), considering that there is often a certain lag in the impacts of economic policy on R&D investment behavior, *EPU*_*t*−1_ with a lag of one period is used to express EPU. The other variables are consistent with the model (4). It should be noted that the EPU index is a macro time series data, the direct introduction of annual virtual variables to control the time effect will cause multiple collinearity, so there is no control time fixed effect in the model. Referring to Wang et al. [27], a macro variable (*M*_*t*_) is added to control the characteristics of the period, and whether EPU will aggravate the peer effects of R&D investment is judged by the positive or negative and significant coefficient *β*_2_.

## 5. Empirical research

### 5.1. Empirical results analysis

First, Empirical results to test the existence of peer effects in R&D investment. Columns (I)-(II) in Table 4 report the regression results of model (4).

**Table 4:** Regression results of models (4) and (5).

Variables	Model(4)		Model(5)	
	(I) *RD*1	(II) *RD*2	(III) *RD*1	(IV) *RD*2
*PeerRD*1	**0.2789** ***		0.1766***	
	**(13.289)**		(3.824)	
*PeerRD*2		**0.2948** ***		0.2361***
		**(14.006)**		(5.030)
*PeerRD*1*EPU			**0.1026** ***	
			**(2.763)**	
*PeerRD*2*EPU				**0.0753** ^**^
				**(1.983)**
*EPU*			-0.0067^**^	-0.0012
			(-2.452)	(-0.966)
*Cash*	0.0036*	-0.0021^**^	0.0035*	-0.0022***
	(1.916)	(-2.568)	(1.853)	(-2.707)
*Lev*	-0.0248***	0.0003	-0.0244***	0.0005
	(-13.203)	(0.352)	(-12.970)	(0.633)
*Roa*	-0.0146***	0.0058***	-0.0126***	0.0071***
	(-4.319)	(3.934)	(-3.751)	(4.840)
*TobinQ*	-0.0002	0.0003***	-0.0006***	0.0001
	(-0.990)	(3.471)	(-2.941)	(0.593)
*Tangible*	0.0043	-0.0025*	0.0055*	-0.0018
	(1.459)	(-1.957)	(1.858)	(-1.400)
*Age*	-0.0025***	-0.0024***	-0.0023***	-0.0022***
	(-4.144)	(-9.030)	(-3.870)	(-8.727)
*Size*	0.0032***	-0.0013***	0.0030***	-0.0015***
	(6.479)	(-6.196)	(5.919)	(-6.850)
*PeerCash*	-0.0250***	-0.0108***	-0.0182**	-0.0056*
	(-3.056)	(-3.059)	(-2.353)	(-1.683)
*PeerLev*	0.0168**	0.0031	0.0158**	0.0049
	(2.159)	(0.921)	(2.085)	(1.481)
*PeerRoa*	-0.0068**	-0.0030**	-0.0048*	-0.0014
	(-2.385)	(-2.423)	(-1.760)	(-1.218)
*PeerTobinQ*	-0.0002	-0.0003***	-0.0004**	-0.0006***
	(-0.852)	(-3.033)	(-2.103)	(-6.977)
*PeerTangible*	-0.0203	-0.0033	-0.0014	0.0066
	(-1.476)	(-0.563)	(-0.107)	(1.161)
*PeerAge*	-0.0071***	0.0000	-0.0068***	-0.0020***
	(-4.568)	(0.015)	(-5.208)	(-3.513)
*PeerSize*	-0.0035**	-0.0011*	-0.0037***	-0.0009
	(-2.531)	(-1.815)	(-2.804)	(-1.584)
ln*M*2			0.0140***	0.0094***
			(7.519)	(11.807)
*eei*			-0.0127***	0.0048***
			(-4.417)	(3.839)
Constant	0.0799**	0.0803***	-0.1043***	-0.0659***
	(2.372)	(5.534)	(-3.625)	(-5.272)
Individual fixed effects	Yes	Yes	Yes	Yes
Year fixed effects	Yes	Yes	No	No
Observations	19931	19931	19931	19931
*R* ^2^	0.840	0.834	0.840	0.832

Note

*, ** and *** are significant at the significance level of 10%, 5% and 1% respectively, and the T-value of regression coefficient is in brackets, the same below.

The explained variable in column (I) is *RD*1, that is, the proportion of total R&D investment in operating revenue; and the explained variable in column (II) is *RD*2, that is, the proportion of total R&D investment in total assets. The coefficient values of *PeerRD*1 and *PeerRD*2 were 0.2789 and 0.2948, respectively, both of which were significant at 1% level. It shows that the R&D input level of peer group enterprises has a significant positive impacts on the R&D input of focus enterprises, that is, when the peer group enterprises have more R&D input in the previous year, the focus enterprises will correspondingly increase the R&D input level of the current year, and vice versa. It can be seen that there is a peer effects on R&D investment based on industry level. Hypothesis 1 is verified.

Then, the impact of EPU on the peer effects of corporate R&D investment. Columns (III)-(IV) of Table 4 report the regression results of model (5), where the explained variable in column (III) is *RD*1, and the explained variable in column (IV) is *RD*2. The regression coefficients of the crossing multiplication term *PeerRD*1*EPU and *PeerRD*2*EPU are significantly positive, indicating that the increase of EPU positively promotes the peer effects of enterprises’ R&D investment. Thus, Hypothesis 2 is verified. That is to say, EPU increases the incompleteness and asymmetry of information obtained by enterprises, and increases the cost of information acquisition.

### 5.2. Endogeneity test

The formulation and adjustment of economic policies belong to macro-control behaviors at the national level, while R&D input belongs to individual decision-making behaviors at the micro level. Individual behaviors are difficult to affect all aspects of economic policies, so there is almost no reverse causality between R&D input decision-making and EPU. In addition, in the construction of the empirical model in this paper, all explanatory variables and control variables are lagged by one period, and the individual fixed effects are strictly controlled, which will avoid the endogeneity problem caused by missing variables to a certain extent. In order to avoid the estimation errors caused by endogeneity problems caused by other possible factors, we refer to Zeng et al. [28] and take the average R&D input (*PeerRD*_*t*−2_) of peer group enterprises lagged by two periods as the instrumental variable of the average R&D input (*PeerRD*1_*t*−1_) of peer group enterprises. Drawing on Peng et al. [29], the global EPU index (*GEPU*_*t*−1_) is used as the instrumental variable of CNEPU index (*EPU*_*t*−1_). Based on the selection of effective instrumental variables, the GMM method is used to estimate models (4) and (5), and the control variables of individual financial characteristics of enterprises and financial characteristics of peer group enterprises are added to the regression (see Table 5). Anderson test (p-value) and Cragg-Donald Wald test (F-value) indicate the validity of the selected instrumental variables.

**Table 5:** Regression results of instrumental variable model.

variables	Model(4)		Model(5)			
	(I) *RD*1_*t*_	(II) *RD*2_*t*_	(III) *RD*1_*t*_		(IV) *RD*2_*t*_	
*PeerRD*1_*t*−1_	**0.4154** ^***^		0.3313***			
	**(9.172)**		(4.426)			
*PeerRD*2_*t*−1_		**0.3863** ^***^			0.2560***	
		**(7.504)**			(2.898)	
*PeerRD*1_*t*−1_*EPU_*t*−1_			**0.0750**			
			**(1.620)**			
*PeerRD*1_*t*−2_*EPU_*t*−1_					**0.1107** ^**^	
					**(2.163)**	
*EPU*_*t*−1_			-0.0081**		-0.0030*	
			(-2.324)		(-1.840)	
The first stage	*PeerRD*1_*t*−1_	*PeerRD*2_*t*−1_	*PeerRD*1_*t*−1_	*EPU*_*t*−1_	*PeerRD*2_*t*−1_	*EPU*_*t*−1_
*PeerRD*1_*t*−1_	0.4437***		0.3452***			
	**(69.300)**		**(33.930)**			
*PeerRD*2_*t*−2_		0.4045***			0.2597***	
		**(58.790)**			**(22.660)**	
*GEPU*_*t*−1_				0. 2363***		0.2352***
				**(137.150)**		**(119.680)**
Control variable	Yes	Yes	Yes		Yes	
Individual fixed effects	Yes	Yes	Yes		Yes	
Year fixed effects	Yes	Yes	No		No	
Andersontest	0.000	0.000	0.000		0.000	
Cragg-Donald test	4802.519	3455.965	1606.903		1052.535	
Observations	17146	17146	17146		17146	
*R* ^2^	0.079	0.079	0.077		0.072	

Note: Due to space limitations, the regression results of the control variables are not reported,similarly below.

It can be seen from column (I) of Table 5 that in the first-stage regression, the average intensity of R&D input (*PeerRD*1_*t*−1_) of peer group enterprises is used as the explained variable, and the coefficient of the instrumental variable, that is, the average intensity of R&D input (*PeerRD*1_*t*−2_) of peer group enterprises lagged by two periods, is 0.4437 and significant at the level of 1%. In the second-stage regression, the explained variable is *RD*1_*t*_, and the core explanatory variable is the average R&D investment intensity (*PeerRD*1_*t*−1_) of enterprises in the same group. The estimated coefficient of *PeerRD*1_*t*−1_ is 0.4154, and it is significant at the level of 1%, indicating that the peer effects of enterprise R&D investment still exists significantly after the endogeneity problem is treated with the instrumental variable method. Column (II) of Table 5 shows the regression results when the R&D investment intensity (*RD*2) is measured in another way. In the regression results of the first and second stages, the coefficient signs and significance levels of the main variables *PeerRD*2_*t*−2_, *PeerRD*2_*t*−1_ are consistent with those in column (I).

It can be seen from column (III) of Table 5 that in the first-stage regression, the coefficients of the instrumental variables, namely the average R&D investment intensity (*PeerRD*1_*t*−2_) of the same group of enterprises lagged by two periods and the global EPU index (*GEPU*_*t*−1_), are significantly positive. The estimated coefficient of *PeerRD*1_*t*−1_*EPU_*t*−1_ in the second-stage regression results is 0.0750, which is approximately significant at the 10% level. Column (IV) of Table 5 shows the regression results when the R&D investment intensity (*RD*2) is measured in another way. In the first-stage regression results, the coefficient signs and significance levels of the main variables *PeerRD*2_*t*−2_ and *GEPU*_*t*−1_ are consistent with those in column (III). Specifically, the estimated coefficient of *PeerRD*2_*t*−1_*EPU_*t*−1_ in the second-stage regression results is 0.1107, which is significant at the 5% level. These results show that the increase of EPU can still enhance the reciprocal effect of enterprise R&D investment by using the instrumental variable method for regression analysis, which once again proves the robustness of the above conclusions.

### 5.3. Robustness test

#### 5.3.1. Change the econometric regression method

In models (4) and (5), considering that the explained variable of the research, namely the data of enterprise R&D investment intensity, is left censored to 0, in order to avoid possible biased and inconsistent results, the benchmark model is replaced by the corresponding Tobit model for estimation. The regression results are shown in columns (I) and (II) of Table 6, and the explained variables are *RD*1. The coefficient of the core explanatory variable *PeerRD*1 of model (4) in column (I) is 0.5374, and the coefficient of the core explanatory variable *PeerRD*1*EPU of model (5) in column (II) is 0.1589, both of which are significant at the level of 1%. It shows that the regression results of the Tobit model still support the previous conclusion that the R&D investment intensity of enterprises will be positively affected by the average R&D investment intensity of the industry, and the positive promoting effect of EPU on the peer effects of enterprise R&D investment is unlikely to be affected by the choice of measurement estimation method.

**Table 6:** Regression results for Tobit model and supplementary missing values of R&D input data.

Variables	Tobit model		supplementary missing values of R&D input data	
	(I) *RD*1	(II) *RD*1	(III) *RD*1	(IV) *RD*1
*PeerRD*1	**0.5374** ^***^	0.3655***	**0.3405** ^***^	0.2310***
	**(29.481)**	(8.198)	**(18.517)**	(5.824)
*PeerRD*1*EPU		**0.1589** ^***^		**0.1052** ^***^
		**(4.338)**		**(3.361)**
*EPU*		-0.0089***		-0.6677***
		(-3.255)		(-3.083)
Control Variables	Yes	Yes	Yes	Yes
Observations	20160	20160	22826	22826

The symbols and significance of core explanatory variables *PeerRD*1 and *PeerRD*1*EPU, are unchanged, which proves the robustness of the conclusion.

#### 5.3.2. Re-measurement of explained variables

China’s Ministry of Finance issued the new Accounting Standards for Business Enterprises in 2006, which requires enterprises to disclose the amount of R&D expenditure. Although it is clearly stipulated that the new accounting standards will be implemented from January 1, 2007, China’s listed companies have a gradual process of adaptation in the disclosure of data related to R&D investment. In fact, Liu et al. [30] pointed out that the proportion of enterprises disclosing R&D expenditure increased significantly in 2011, so it is reasonable to believe that the implementation of the new accounting standards has been quite effective. Therefore, if the samples with missing R&D investment are discarded across the board, it may lead to the loss of observations or sample selection bias.

In order to alleviate this problem, referring to the practice of Liu et al. [30], the samples of R&D expenditure from 2008 to 2011 that are shown as vacancies in the annual reports are treated as missing values. If the R&D expenditure data of enterprises in the annual reports of 2012 and subsequent years are still vacant, it can be reasonably supplemented as 0. These samples are supplemented to the original regression samples to verify the robustness of the above results. The regression results of models (4) and (5) are shown in columns (III) and (IV) in Table 6.

#### 5.3.3. Changing the calculation method of core explanatory variables

When measuring the R&D investment intensity *PeerRD* of the same group of enterprises above, the average value of the R&D investment intensity of other enterprises in the same industry after the focal enterprise is removed is calculated. Referring to Zeng et al. [28], the robustness test is carried out by measuring *PeerRD* using the median value of R&D investment intensity of other enterprises in the same industry after the focal enterprise is removed. At the same time, the calculation method of the control variables of the financial characteristics of the same group of enterprises is also changed from the corresponding variable industry-annual mean to the median. The results of models (4) and (5) after re-regression are shown in columns (I) and (II) of Table 7.

**Table 7:** Regression results after changing variable measurement methods.

Variables	Change the measurement method of R&D input of enterprises in the same group		Change the measurement method of economic policy uncertainty	
	(I) *RD*1	(II) *RD*1	(III) *RD*1	(IV) *RD*1
*PeerRD*1	**0.3327** ^***^	0.0937	0.1573***	0.2591***
	**(12.019)**	(1.605)	(3.053)	(10.727)
*PeerRD*1*EPU		**0.2077** ^***^	**0.1224** ^***^	**0.0201** ^**^
		**(4.594)**	**(2.824)**	**(2.348)**
*EPU*		-0.0144***	-0.0060*	-0.0018***
		(-5.269)	(-1.933)	(-3.245)
Control Variables	Yes	Yes	Yes	Yes
Individual Fixed Effects	Yes	Yes	Yes	Yes
Year fixed effects	Yes	No	No	No
Observations	19931	19931	19931	19931
*R* ^2^	0.840	0.840	0.840	0.840

In addition, in order to further test the robustness of the construction method of EPU index, firstly, we try to use the median value method to re-convert the monthly economic uncertainty index into the annual economic uncertainty index. The regression results of model (5) are shown in column (III) of Table 7. Secondly, the EPU index constructed by Baker et al. [17] is used to measure the degree of EPU. The regression results of model (5) are shown in column (IV) of Table 7. In the regression results in columns (I)—(IV) of Table 7, the signs and significance of the core explanatory variables are basically unchanged, which again proves the robustness of the conclusions.

## 6. Further examination

### 6.1. Based on the motivation of enterprise managers to maintain reputation

The influence of EPU on enterprise development may be channeled through the behavioral decisions of managers. Scharfstein & Stein [31] argued that, the labor market for manager of a company is completely competitive, but there is information asymmetry between the company (labor demand side) and the manager. To ensure that managers’ personal reputations are at market average, managers will choose to mimic the behavior of managers at peer companies rather than make decisions based on their own private information (which increases the risk of personal reputation). Gao et al. [15] concluded that managers often adopt similar tax avoidance decisions as other business managers in order to maintain their professional reputation in the industry, ensure their own performance, and win the favor of those who need employment. Under the environment of EPU, the degree of information incompleteness and asymmetry increases, which increases the risk that managers have to bear in R&D investment decision-making, and makes it more difficult and cost for managers to obtain the information needed for R&D investment decision-making. Therefore, in order to maintain their professional reputation, enterprise managers with bounded rationality are more inclined to be close to the average level of the industry, rather than make decisions based on their own private information. In this process, the organizational form with the separation of the two rights provides the possibility for managers’ self-interested behaviors. Choosing the trend and intensity of R&D investment that is basically consistent with most firms in the industry can mask the risks that managers have to bear in their R&D investment decisions. If the decision is successful, managers will be regarded as wise and intelligent. Even if the R&D investment project fails, it is not only the company that suffers losses, and managers are more likely to attribute it to external uncontrollable factors rather than their own ability. In other words, it is better for the manager’s reputation to fail in the conventional way than to succeed in the unconventional way. Therefore, the more attention managers attach to their professional reputation, the more obvious the peer effects of corporate R&D investment may be under the EPU. In order to test the moderating role of managers’ motivation to maintain reputation in the process of EPU affecting the peer effects of R&D investment, CEO’s age (logarithmic treatment), CEO’s tenure and executive salary (logarithmic treatment) are used to measure managers’ motivation to maintain reputation and avoid risks from multiple perspectives. The median values of the three variables were calculated respectively according to the industry, and the samples were divided into sub-sample groups with different reputation maintenance motivations by using the median as the boundary. Then, regression was carried out respectively according to model (5). The results are shown in Table 8.

**Table 8:** Channel test based on managers’ motivation to maintain reputation.

variables	(I)	(II)	(III)	(IV)	(V)	(VI)
	Ceos are older	Ceos are young	Long CEO tenure	Short CEO tenure	Low executive pay	High executive pay
*PeerRD*1	0.3153***	0.0203	0.2127***	-0.6127***	0.2406***	0.0534
	(3.674)	(0.225)	(4.009)	(-3.756)	(3.292)	(0.902)
*PeerRD*1*EPU	**-0.0709**	**0.2696** ^***^	**0.0776** ^*^	**0.8514** ^***^	**0.0028**	**0.1674** ^***^
	**(-0.914)**	**(3.222)**	**(1.922)**	**(5.365)**	**(0.046)**	**(3.731)**
*EPU*	-0.0044	-0.0078	-0.0093***	-0.0104	0.0014	-0.0105***
	(-0.898)	(-1.504)	(-3.007)	(-1.249)	(0.317)	(-3.116)
Control Variables	yes	yes	yes	yes	yes	yes
Individual Fixed Effects	yes	yes	yes	yes	yes	yes
Observations	8171	9036	14893	4497	9723	9632
*R* ^2^	0.872	0.871	0.865	0.838	0.830	0.901

In Table 8, the explained variables corresponding to all regressions are, and control variables at the individual level and the peer level as well as macroeconomic variables are added. The samples in columns (I) and (II) of Table 8 correspond to the groups with older ceos and older ceos, respectively. Compare the coefficients of the cross-multiplication terms *PeerRD*1*EPU in columns (I) and (II), It changes from insignificant −0.0709 to significant 0.2696 at the level of 1%, indicating that EPU plays a greater role in promoting peer effects in the sample group with younger CEOs than in the sample group with older CEOs. Young managers at the early stage of their careers, without rich experience and facing the uncertainty of external economic policies, are more inclined to make a sound R&D investment decision that is convenient to shift responsibility once the project fails.

The samples in columns (III) and (IV) of Table 8 respectively correspond to the groups with longer and shorter CEO tenure. Comparing the coefficient of the interaction term *PeerRD*1*EPU in columns (III) and (IV), it changes from 0.0776 to 0.8514. This indicates that EPU has a greater role in promoting peer effects in the sample group with a shorter CEO tenure than in the sample group with a longer CEO tenure. This shows that managers at the initial stage of their career show stronger peer effects on R&D investment when facing greater uncertainty in the external environment.

The samples in columns (V) and (VI) of Table 8 correspond to the groups with low and high executive compensation, respectively. Comparing the coefficient of the interaction term *PeerRD*1*EPU in columns (V) and (VI), it increases from 0.0028 to 0.1674, and changes from insignificant to significant at the level of 1%, indicating that the aggravating impacts of EPU on peer effects is more significant in the sample group with higher salary. This shows that in order to maintain professional reputation and maintain the current salary level, managers of enterprises with higher salary tend to refer to the decision-making of enterprises in their peer group to avoid risks and reduce performance fluctuations when EPU is large.

### 6.2. Based on the mediating effect of corporate financing constraints

High EPU will exacerbate the degree of market information asymmetry, lead to the increase of financial friction, reduce the availability of corporate credit resources, and increase the financing cost of enterprises (see Li et al. [32]). Specifically, on the one hand, for the equity financing of enterprises, the rise of uncertainty will lead to the fluctuation of the stock market (see Baker et al. [17]), resulting in the increase of the risk level in the capital market. On the other hand, for corporate debt financing, the rise of EPU increases the friction in the financial market, and banks and other financial intermediaries show a certain degree of "loan reluctance" in order to avoid risks (see Baum et al. [33]). In addition, the increase of information asymmetry reduces the commercial credit between upstream and downstream enterprises in the market. All these aspects have increased the difficulty and cost of corporate debt financing and increased the level of corporate financing constraints (see Francis et al. [34]).

Enterprise R&D innovation activities have high technical content, and it is a long and complex process from R&D investment to innovation output and then to product marketing to bring corporate profits, which requires higher capital and human input than general investment. Firms with higher degree of financing constraints are at an information disadvantage in the market, with less information related to R&D decisions and weaker ability to resist risks in the fierce market competition. Against the background of increasing macroeconomic policy uncertainty, high financing constraints make enterprises tend to keep consistent with the behavior trend of most enterprises in order to avoid risks. Therefore, firms with high financing constraints may also be more inclined to learn from other firms in the same industry, thus strengthening the peer effects of firm R&D investment.

Based on the above analysis, this paper concludes the influence path of "EPU—financing constraint—peer effects of R&D investment". In order to test the mediating role of corporate financing constraints in the process of EPU affecting the peer effects of R&D investment, Wen et al. [35] further constructed models (6) and (7) by adding mediating variables *SA* on the basis of Model (5):

$$SA_{ijt-1}=\alpha +\beta_{1}EPU_{t-1}+\gamma_{1}Controls_{ijt-1}^{Firm}+\gamma_{2}Controls_{-ijt-1}^{Peer}+\mu_{i}+\theta M_{t}+\varepsilon_{it}$$ (6)

$$\begin{array}{l} RD_{ijt}=\alpha +\beta_{1}PeerRD_{-ijt-1}+\beta_{2}PeerRD_{-ijt-1}\times EPU_{t-1}+\beta_{3}EPU_{t-1}+\beta_{4}SA_{ijt-1}\\ \;+\beta_{5}PeerRD_{-ijt-1}\times SA_{ijt-1}+\gamma_{1}Controls_{ijt-1}^{Firm}+\gamma_{2}Controls_{-ijt-1}^{Peer}+\mu_{i}+\theta M_{t}+\varepsilon_{it} \end{array}$$ (7)

Where the variable *SA* represents the level of corporate financing constraints. Referring to Hadlock & SPierce [36] and Ju et al. [37], the index is used to represent the financing constraint level of the enterprise. The calculation method is: $\mathrm{abs}(-0.737\times Size+0.043\times Size^{2}-0.04\times Age)$, Among them, *Size* is the natural logarithm of the total assets of the enterprise (unit: million yuan), *Age* is the age of the enterprise.

The regression results of models (5), (6) and (7) are shown in columns (I), (II) and (III) of Table 9, respectively, where the R&D investment intensity variable is *RD*1.

**Table 9:** EPU, financing constraints and the peer effects of R&D investment.

variables	(I) *RD*1	(II) *SA*	(III) *RD*1	(IV) *RD*1	(V) *RD*1
*PeerRD*1	0.1766***		-0.1797	0.0443	0.1870***
	(3.824)		(-1.164)	(0.739)	(2.624)
*PeerRD*1*EPU	**0.1026** ^***^		**0.0515**	**0.1919** ^***^	**0.0700**
	**(2.763)**		**(1.231)**	**(3.955)**	**(1.230)**
*EPU*	-0.0067**	**0.0541** ^***^	-0.0052*	-0.0112***	-0.0064
	(-2.452)	**(13.680)**	(-1.839)	(-3.146)	(-1.641)
*PeerRD*1*SA			**0.1137** ^**^		
			**(2.454)**		
*SA*			-0.0013		
			(-0.326)		
Control Variables	yes	yes	yes	yes	yes
Individual Fixed Effects	yes	yes	yes	yes	yes
Observations	19931	19931	19931	9818	9893
*R* ^2^	0.840	0.983	0.840	0.858	0.872

It can be seen from column (I) of Table 9 that the coefficient *β*_2_ of the variable *PeerRD*1*EPU in model (5) is significantly positive, indicating that *EPU* exacerbates the peer effect of corporate R&D investment. It can be seen from column (II) of Table 9 that the regression coefficient *β*_1_ of the explanatory variable *EPU* on the explained variable *SA* in Model (6) is 0.0541, which is significant at the level of 1%, indicating that EPU has a significantly positive promoting effect on the mediating variable. Furthermore, in column (III) of Table 9, the coefficient *β*_5_ corresponding to the interaction term *PeerRD*1*SA in Model (7) is 0.1137, which is significant at the level of 1%, indicating the existence of the mediating effect. At the same time, the coefficient *β*_2_ of the variable *PeerRD*1*EPU reflects the influence on the peer effects of the enterprise from *EPU*, which is 0.1026 in the regression result of model (5), that is, column (I) of Table 9, and 0.0515 in the regression result of model (7), that is, column (III) of Table 9. This paper further explains the existence of the intermediary effect with financing constraints as the intermediary variable, and again provides empirical evidence for the influence of EPU on the behavioral decisions of micro enterprises through the channel of financial friction.

In order to further test the role of corporate financing constraints in the process of EPU influencing the peer effect of R&D investment, the median of corporate financing constraints was calculated by industry and year by year, and the samples were divided into two sub-sample groups: high and low financing constraints. Then, regression was carried out respectively according to model (5). The results are shown in columns (IV)—(V) of Table 9, and the explained variables are *RD*1. In the sample group with high degree of financing constraints, the estimated coefficient of the cross-multiplication term *PeerRD*1*EPU is 0.1919, and it is significant at the level of 1%. In the other group of samples corresponding to the low degree of financing constraints, the estimated coefficient of *PeerRD*1*EPU is only 0.0700 and the significance is poor. This indicates that EPU has a greater role in promoting peer effects in the sample group with a higher degree of financing constraints than in the sample group with a lower degree of financing constraints, which is essentially consistent with the conclusion in column (III).

## 7. Conclusions and policy recommendations

In this paper, LDA machine learning method is used to extract the text topics of newspaper news and construct the Chinese EPU index. On this basis, based on the relevant data of A-share listed companies from 2009 to 2020, this paper empirically tests the existence of the peer effects of listed firms R&D input, and further analyzes the impacts of EPU on the peer effect of enterprises’ R&D input. The conclusions are as follows:

Firstly, there is a peer effects at the industry level, that is, when making R&D investment decisions, Chinese listed enterprises will learn and imitate the R&D investment decisions of other enterprises in the same industry.

Secondly, the greater the degree of EPU, the more obvious the peer effects of R&D investment. In order to verify the reliability of the conclusion, this paper uses instrumental variable method, Tobit model, replacement of explained variables and core explanatory variables to further test robustness, and the conclusion remains unchanged. Further research shows that the younger the manager is, the shorter the tenure, and the higher the salary, that is, the stronger the motivation of the manager to maintain reputation and avoid risks, the more obvious the positive impacts of EPU on the peer effects of R&D investment, which indicates the moderating effect of the motivation of the manager to maintain reputation on the peer effects of the EPU.

Finally, it verifies the mechanism that EPU affects the peer effects of R&D input through financial friction.

Based on theoretical analysis and empirical results, this paper tries to put forward the following policy suggestions:

One hand, for enterprises, imitation and learning among peers can affect the level of R&D investment of enterprises, and the level of R&D investment of enterprises is directly related to the innovation efficiency and profitability of enterprises in the future. Blindly following and imitating may lead to inefficient R&D investment. Therefore, enterprises should pay attention to improving managers’ ability and carefully identify R&D information transmitted by their peers with professional skills. On the basis of accurately grasping the government’s R&D policies and the future market development prospects, we should make reasonable decisions that not only learn from others but also consider our own actual conditions.

On the one hand, the peer effects originate from enterprises’ demand for decision-making information on R&D investment of the same industry, and the government is also important for enterprises’ information acquisition. The EPU will increase the cost and imperfection of information acquisition by enterprises themselves. Therefore, the government should pay attention to maintaining communication and exchange with enterprises, establish a multi-channel information disclosure platform, release real and reliable research and development trends, and ensure the robustness, integrity and consistency of economic policies formulated, guide enterprises to form rational expectations, so as to improve the efficiency of enterprise research and development investment.

## Supporting information

S1 File Supporting information containing S1-S5 Data and S6 Code. (ZIP)
