# Supplementary material for: The impacts of economy policy uncertainty on peer effects of firms R&D investment: Based on LDA machine learning and regression statistical modeling approach
Source: PLoS One. 2024 Jun 24;19(6):e0305715. doi: 10.1371/journal.pone.0305715 (PMC11195977; doi:10.1371/journal.pone.0305715)
Supplement: S1 File — (ZIP) [file pone.0305715.s001.zip › data&code/supporting file.docx]

**basicdata.dta**

**basicdata_supplementary.dta**

**CEO_age.dta**

**CEO_tenure.dta**

**executive_pay.dta**

**code.do**
